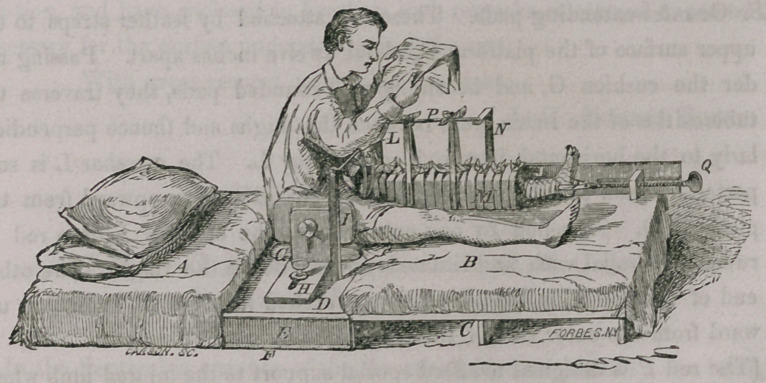# The Burges’ Thigh Splint, or Fracture Bed

**Published:** 1858-11

**Authors:** Frank H. Hamilton

**Affiliations:** Buffalo


### ART. II.— *The Burges' Thigh Splint, or Fracture Bed.* By Frank H. Hamilton, M. D., Buffalo.

Dear Doctor: Accompanying this note you will find a couple of electrotypes, with also a complete description of them. They are intended to explain the thigh splint, or fracture bed of the Burges' (J. H. Hobart Burge and Brother,) of Brooklyn, N. Y. It is the same apparatus of which Dr. Mott speaks in terms of commendation in a letter addressed to me, and which letter you will find published in the third part of my "Report on Deformities after Fractures," at page 317 of the tenth volume of the Transactions of the American Medical Association. These gentlemen having furnished me with a complete apparatus, I have, myself, had one opportunity of giving it a trial in the case of an oblique fracture of the femur in an adult laboring man. The limb has united with a shortening of not more than half an inch; and this can only be detected by a careful examination with a tape-line. He walks without any halt. This result is quite equal to any which I have ever obtained in similar cases; and I am confident that surgeons will find this apparatus a valuable addition to their means of reduction and retention in cases of broken femurs.

The apparatus is now in use at some of the New York hospitals, and has been favorably noticed by several excellent surgeons.

It will be seen, by a inspection of the drawings, that some improvements have been made upon the apparatus since the publication of my report in the Transactions, (pp. 440—41, appendix.)

For a more full account of the fracture bed you may consult the New York Jour. of Medicine for May, 1857.

The price of the improved bed and splint is $35.

At my request these descriptions have been furnished for your Journal; and I sincerely hope that surgeons will be soon pursuaded to abandon their rickety double-inclined planes in cases of broken femurs, and substitute either this or some other form of straight splint. Considering that this is both a bed and a splint it must be regarded as cheap, and, perhaps, as the cheapest suitable apparatus which can be supplied.

If any surgeon thinks that a shortening of half an inch in cases of oblique fracture in adults implies that their apparatus is faulty, then he will continue to employ such as his experience has proven can furnish better results; but I am very much afraid that he is deceiving himself, and that he will not discover his error until some medical friend measures the limb for him, and some court of law metes to him according to his measure.

Very truly yours,

FRANK H. HAMILTON.

AUSTIN FLINT, Jr.

*A.* Thick mattress.

*B.* Thin mattress.

*C.* Wooden platform upon which the thin mattress is laid.

(This platform is made in two pieces and hinged together so as to fold upon itself for convenience of transportation, and when in use is merely hooked upon the central platform D.)

*D.* Central or cushioned platform supported at either end by wooden strips marked E. which rest upon

*F.* A second platform of same extent as D.

(This constitutes a shelf for the bed pan, which may be introduced below from either side.)

*G.* Firm, but easy hair cushion, upon which the hips of the patient rest.

(This cushion, as well as the platform D, to which it is buttoned, has a semicircular opening at its lower margin for convenience of defecation.)

*H.* A rectangular wooden slide, exactly corresponding to its fellow upon the opposite side of the pelvis. These slides are so arranged upon the platform D as to be separated or approximated at will, and, by a thumb screw which passes through a fissure in the horizontal portion of each, they may be fixed at the desired point so as exactly to embrace the pelvis of any patient. There is also a fissure in the perpendicular portion of each rectangular slide, and a screw passing through the same. One of these is to secure the upper end of the long splint J, and the other for the attachment of a short splint I, upon the side of the pelvis corresponding to the uninjured limb. Both of these splints are well padded upon one surface and may be elevated or depressed at will, in order to bring them to the level of the limbs, and fixed at the proper attitude by the screws already mentioned. They are also mutually transferable, thus adapting the apparatus to fractures of either thigh.

*SS.* Counter-extending pads. These are attached by leather straps to the upper surface of the platform D, about twelve inches apart. Passing under the cushion G, and becoming well rounded pads, they traverse the tuberosities of the ischia, pass between the thighs and thence perpendicularly to the horizontal iron rod or *crossbar* L. The crossbar L is supported at each end by a perpendicular bar extending upward from the platform D. Attached by one extremity to the crossbar L, is a rod P, running parallel with and situated directly above the thigh. The other end of this rod P, is supported by an arched iron bar N, extending upward from the outer side of the long splint J.

(The rod P is designed to afford special support to the injured limb whenever such support is deemed advisable, and is, we think, in many cases of essential service in preserving the arched form of the femur. Two or three strips of cotton cloth, of suitable width, may be passed around the limb, either internally or externally to the splints of coäptation, and tied over the supporting rod P. Splints of coäptation are to be applied according to the exigencies of the case.)

M. An inside splint covered by the bandages.

(The dressings, in one respect not being well represented in the engraving, it seems necessary to say at this point that we generally apply the *outside* bandage in the following manner. Place four or five strips of cotton cloth—two and a-half inches wide and six or eight feet long—transversely under the limb, a short distance apart. In relation to the long external splint, and also to the internal splint, these strips should first pass between them and the limb; secondly, they should be reflected over these splints and pass downward upon the outside of each; thirdly, they should be crossed beneath the limb, and if a posterior splint be used, beneath this also; fourthly, bring the ends of each bandage up upon opposite sides of the limb, outside of all the splints and tie them over the limb, and, if an anterior splint be used, outside this also.)

Q. The screw by which extension is effected in the ordinary way.

(It has at one extremity a swivel and hook tied to a strip of wood in the loop of adhesive plaster below the foot. The ends of the strip of plaster extend upon either side of the limb to near the point of fracture, being kept in place by a roller bandage evenly and rather firmly applied from the toes.)

Brooklyn, Sept. 10th, 1858.

Dear Doctor,—The electrotypes are entirely at your service for whatever use you may please to make of them. I send herewith the promised *description*, and have enclosed in brackets such remarks as seemed to me to be necessary for the perfect understanding of the rest.

With great respect, your obed't servant,

J. H. Hobart Burge.

## Figures and Tables

**Figure f1:**
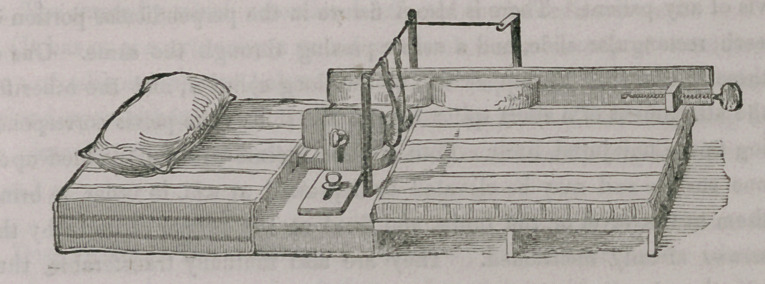


**Figure f2:**